# Supplementary material for: Alpha Carbonic Anhydrase from Nitratiruptor tergarcus Engineered for Increased Activity and Thermostability
Source: Int J Mol Sci. 2024 May 28;25(11):5853. doi: 10.3390/ijms25115853 (PMC11173315; doi:10.3390/ijms25115853)
Supplement: Supplementary file 1 [file ijms-25-05853-s001.zip › ijms-2989948-supplementary.pdf]

SUPPLEMENTARY DATA

Supplementary Table S1. Protein sequence of *Nitratiruptor tergarcus* carbonic anhydrase *(*NtCA) and primers used for recombinant CA construction. In italics are the residues making up the signal peptide which were removed during cloning. Residues in bold and underline are mutations.

Gene	Protein sequence	Forward Primer (5'-3')	Reverse Primer (3'-5')
NtCA	*MKKVFVIAGILTGLLFAGVSGEGHH*AEHKHWGYSGSEGPKHWGEIDPKFRMCKFGVNQSPVDMTRFIEAKLPKLQIIYAGISQNVVNNGHTIKVTTQGKNEVVVDGIPFVLLQYHFHTPSENTINGKHFPMEAHFVHKSKDGEYLVIALMFKEGKKNRALEKVLTYLDPKVGHKQPLKEMFNPGDFFPKKLDYYRYDGSFTTPPCTEGVRWIVLKNAVEASKEQIAKMHKIMGSNNRPTQPLHARVILK	TAAGAAGGAGATATACCATGGCGGAGCATAAACATTGGGG	CAGTGGTGGTGGTGGTGGTGCTTTAATATCACGCGGGCGT
Q1 (A159F, D168P, D197L, R210L)	AEHKHWGYSGSEGPKHWGEIDPKFRMCKFGVNQSPVDMTRFIEAKLPKLQIIYAGISQNVVNNGHTIKVTTQGKNEVVVDGIPFVLLQYHFHTPSENTINGKHFPMEAHFVHKSKDGEYLVIALMFKEGKKNR**F**LEKVLTYL**P**PKVGHKQPLKEMFNPGDFFPKKLDYYRY**L**GSFTTPPCTEGV**L**WIVLKNAVEASKEQIAKMHKIMGSNNRPTQPLHARVILK	TAAGAAGGAGATATACCATGGCGGAGCATAAACATTGGGG	CAGTGGTGGTGGTGGTGGTGCTTTAATATCACGCGGGCGT

Supplementary Table S2. Sequence of primers used for NtCA site directed mutation. In bold and underlined are the codons of the mutant residue.

Mutant	Template	Forward Primer 2 (5'-3')	Reverse Primer 2 (3'-5')
N88K	NtCA	AAAATGTTGTAAAC**AAA**GGCCACACTATCAAAGTAACG	ACTTTGATAGTGTGGCC**TTT**GTTTACAACATTTTGCGATA
N88H	NtCA	AAAATGTTGTAAAC**CAT**GGCCACACTATCAAAGTAAC	TTGATAGTGTGGCC**ATG**GTTTACAACATTTTGCGAT
K138L	NtCA	TCATTTTGTTCAT**CTG**AGCAAAGACGGTGAGTATCTG	CACCGTCTTTGCT**CAG**ATGAACAAAATGAGCTTCCAT
Y144I	NtCA	GCAAAGACGGTGAG**ATT**CTGGTAATTGCCTTAATGT	AGGCAATTACCAG**AAT**CTCACCGTCTTTGCTCTTATG
Y144L	NtCA	GCAAAGACGGTGAG**CTG**CTGGTAATTGCCTTAATGTTT	AGGCAATTACCAG**CAG**CTCACCGTCTTTGCTCTTA
A159D	NtCA	GAAAGAAGAATCGC**GAC**TTAGAGAAGGTTTTAACT	AAAACCTTCTCTAA**GTC**GCGATTCTTCTTTCCTTC
A159F	NtCA	GGAAAGAAGAATCGC**TTT**TTAGAGAAGGTTTTAACTTAT	GTTAAAACCTTCTCTAA**AAA**GCGATTCTTCTTTCCTTCT

D168P	NtCA	GTTTTAACTTATCTT**CCG**CCAAAAGTAGGGCATAAGC	GCCCTACTTTTGG**CGG**AAGATAAGTTAAAACCTTCTC
D168K	NtCA	TTTAACTTATCTT**AAA**CCAAAAGTAGGGCATAAGCAA	TGCCCTACTTTTGG**TTT**AAGATAAGTTAAAACCTTC
D197L	NtCA	ATTACTACCGGTAC**CTG**GGTTCGTTCACCACCCC	TGGTGAACGAACC**CAG**GTACCGGTAGTAATCCAG
D197E	NtCA	TACTACCGGTAC**GAA**GGTTCGTTCACCACCCCCC	GGTGGTGAACGAACC**TTC**GTACCGGTAGTAATCCAG
R210L	NtCA	GCACCGAAGGGGTC**CTT**TGGATAGTTTTAAAGAAT	TTAAAACTATCCA**AAG**GACCCCTTCGGTGCAAGGG
R210K	NtCA	CACCGAAGGGGTC**AAA**TGGATAGTTTTAAAGAATGCTG	CTTTAAAACTATCCA**TTT**GACCCCTTCGGTGCAAGGGG
A159F, R210L	A159F	GCACCGAAGGGGTC**CTT**TGGATAGTTTTAAAGAAT	TTAAAACTATCCA**AAG**GACCCCTTCGGTGCAAGGG
A159F, R210K	A159F	CACCGAAGGGGTC**AAA**TGGATAGTTTTAAAGAATGCTG	CTTTAAAACTATCCA**TTT**GACCCCTTCGGTGCAAGGGG
N88K, R210L	N88K	GCACCGAAGGGGTCCTTTGGATAGTTTTAAAGAAT	TTAAAACTATCCAAAGGACCCCTTCGGTGCAAGGG
N88K, R210K	N88K	CACCGAAGGGGTC**AAA**TGGATAGTTTTAAAGAATGCTG	CTTTAAAACTATCCA**TTT**GACCCCTTCGGTGCAAGGGG
D168K, R210L	D168K	GCACCGAAGGGGTCCTTTGGATAGTTTTAAAGAAT	TTAAAACTATCCAAAGGACCCCTTCGGTGCAAGGG
D168K, R210K	D168K	CACCGAAGGGGTC**AAA**TGGATAGTTTTAAAGAATGCTG	CTTTAAAACTATCCA**TTT**GACCCCTTCGGTGCAAGGGG
Q2 (A159F, D168P, D197E, R210L)	Q1	TACTACCGGTAC**GAA**GGTTCGTTCACCACCCCCC	GGTGGTGAACGAACC**TTC**GTACCGGTAGTAATCCAG
Q3 (A159F, D168P, D197L, R210K)	Q1	CACCGAAGGGGTC**AAA**TGGATAGTTTTAAAGAATGCTG	CTTTAAAACTATCCA**TTT**GACCCCTTCGGTGCAAGGGG
Q4 (A159F, D168P, D197E, R210K)	Q2	CACCGAAGGGGTC**AAA**TGGATAGTTTTAAAGAATGCTG	CTTTAAAACTATCCA**TTT**GACCCCTTCGGTGCAAGGGG

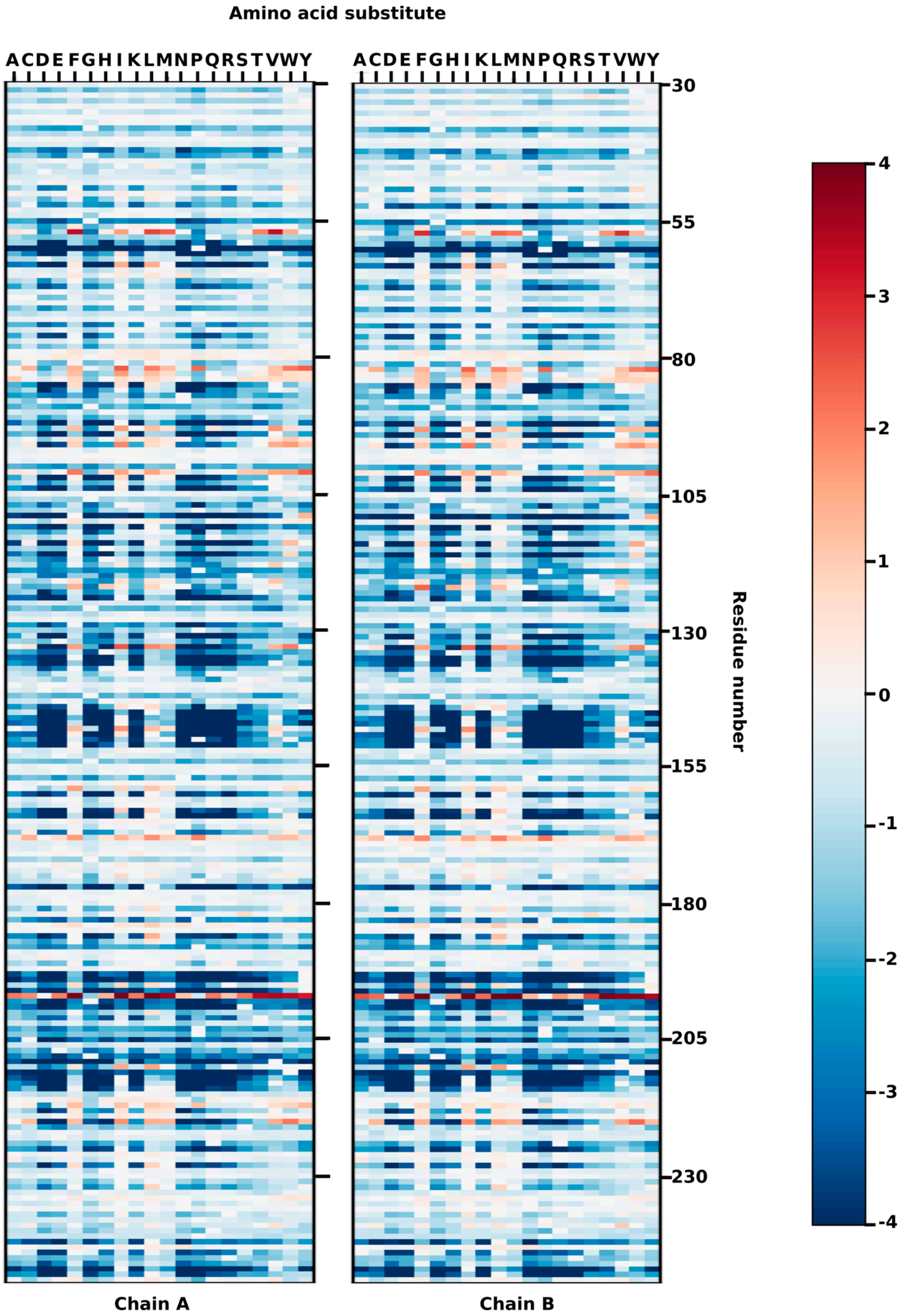

Supplementary Figure S1. Heatmap of ΔΔG values of all possible single mutations in chains A and B of NtCA. Values more than 0 suggest a more stable variant and values less than 0 suggest a less stable variant.

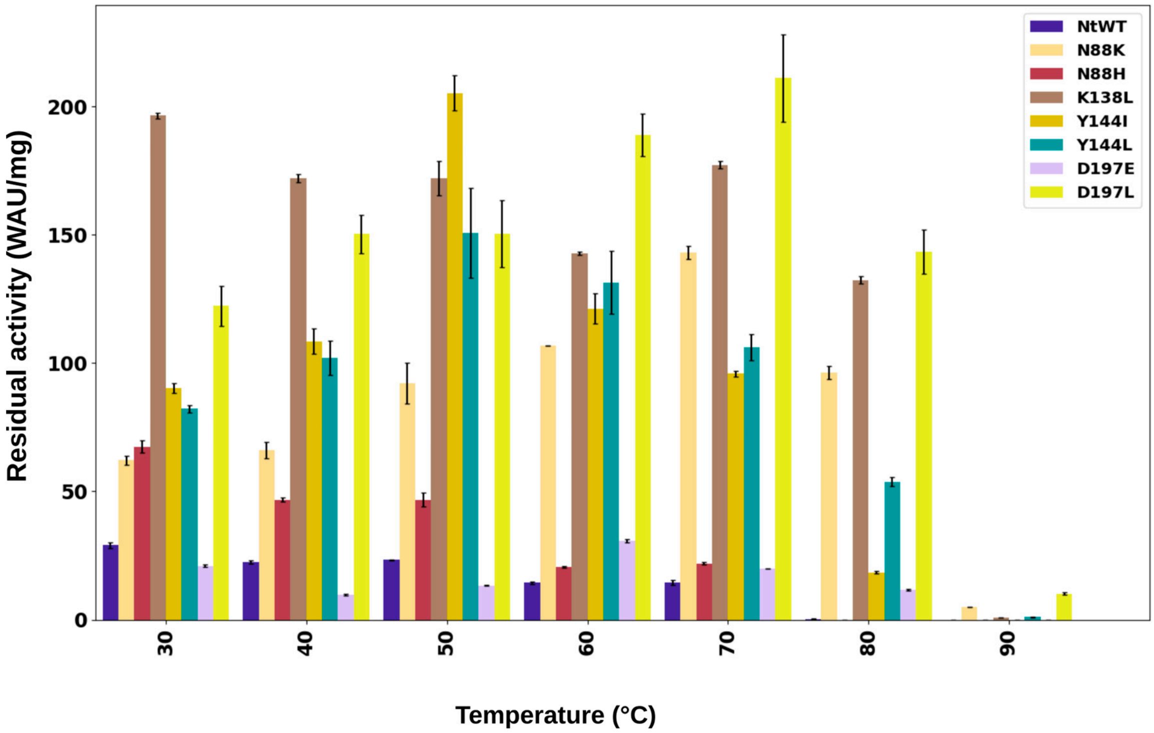

Supplementary Figure S2. Residual activity of NtCA and single mutants after incubation for 1 hr at 30 °C – 90 °C.

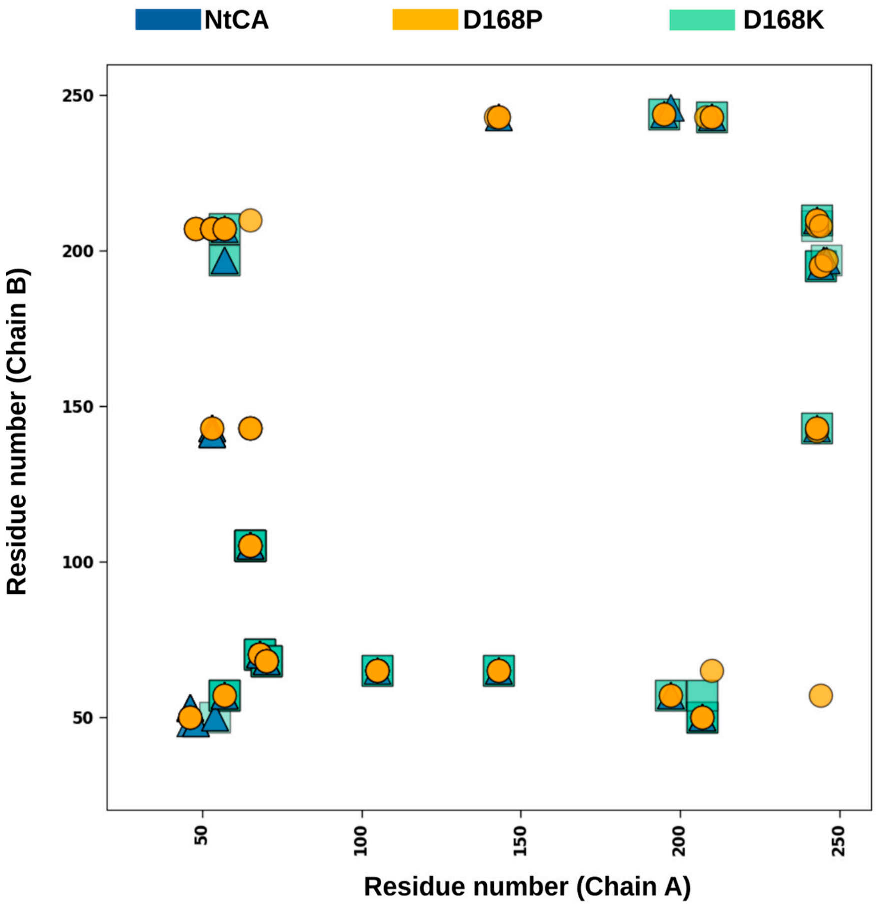

Supplementary Figure S3. Interface hydrogen bond scatter plot showing bonds formed between chain A and chain B of NtCA (blue triangles), D168P (orange circles) and D168K (light green squares).

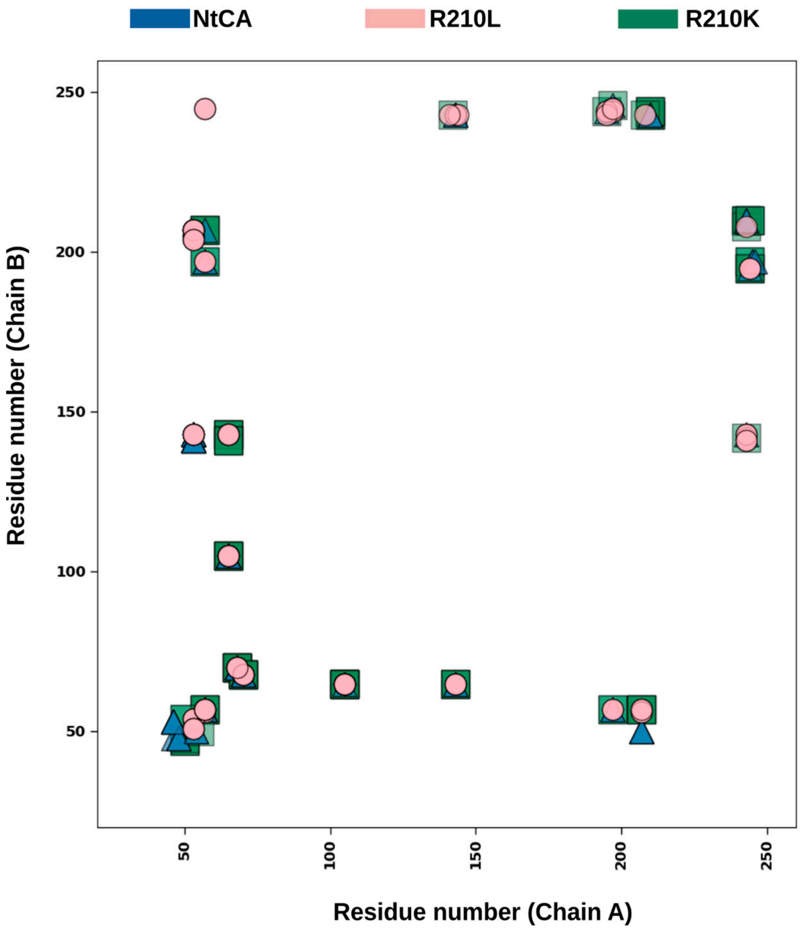

Supplementary Figure S4. Interface hydrogen bond scatter plot showing bonds formed between chain A and chain B of NtCA (blue triangles), R210L (pink circles) and R210K (green squares).

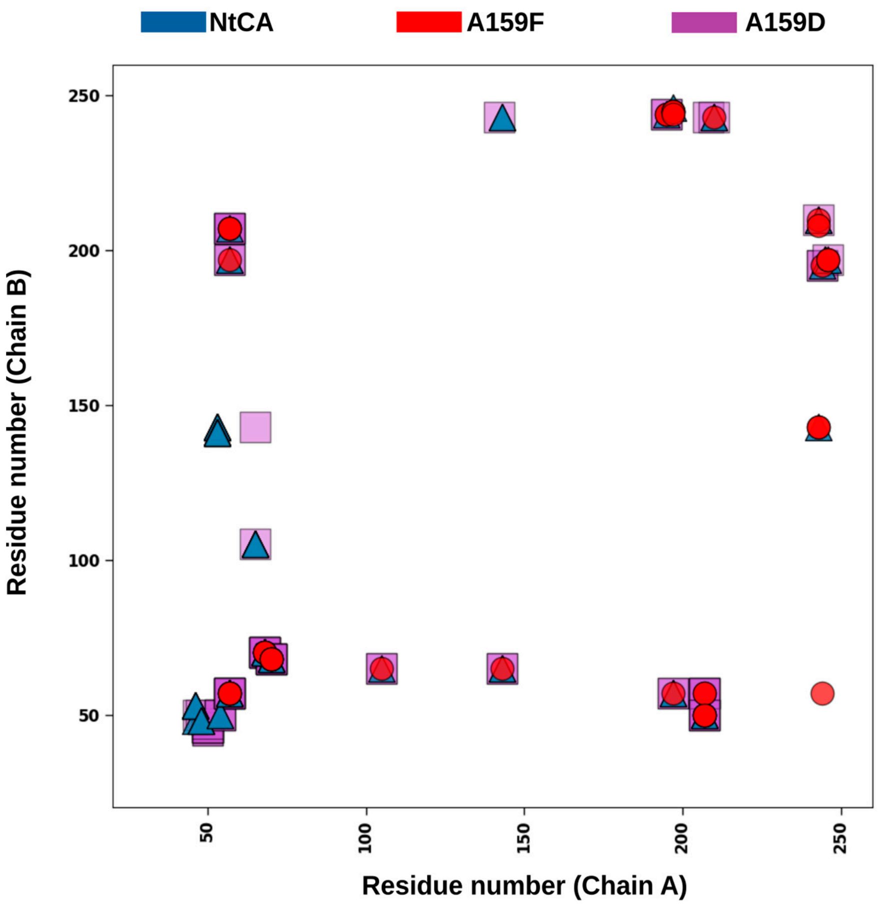

Supplementary Figure S5. Interface hydrogen bond scatter plot showing bonds formed between chain A and chain B of NtCA (blue triangles), A159F (purple circles) and A159D (purple squares).

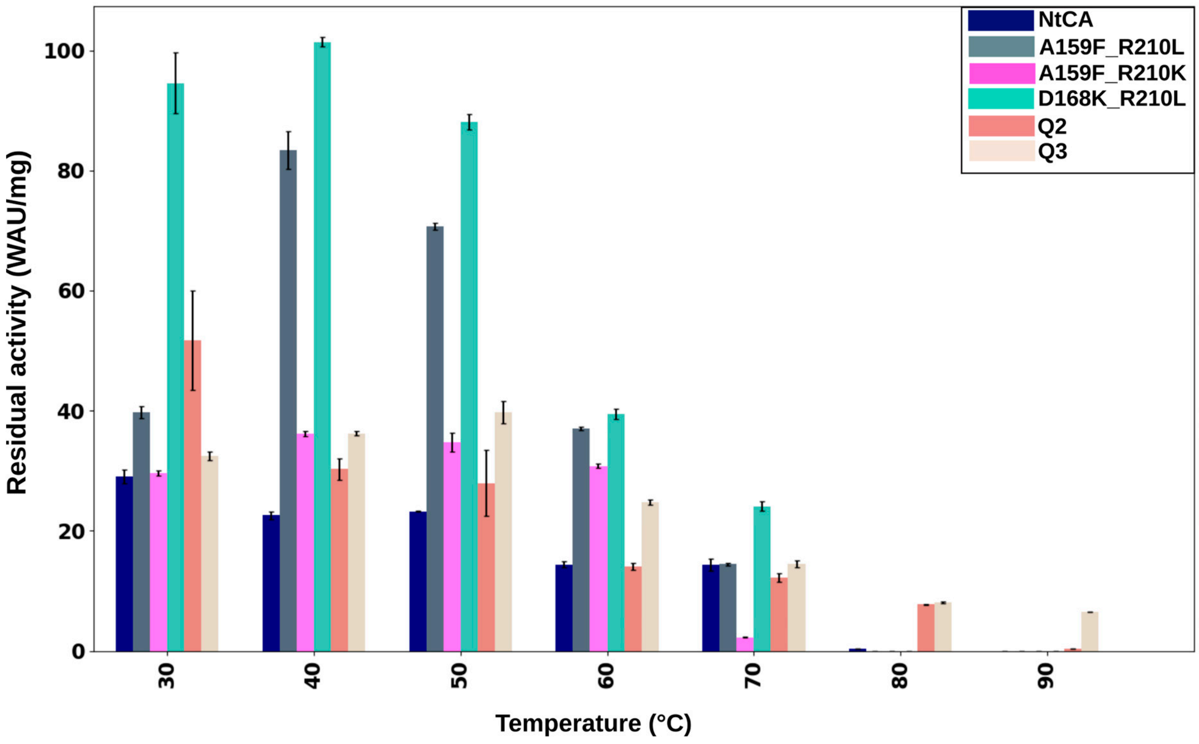

Supplementary Figure S6. Residual activity of NtCA and combinations mutants after incubation for 1 hr at 30 – 90 °C.

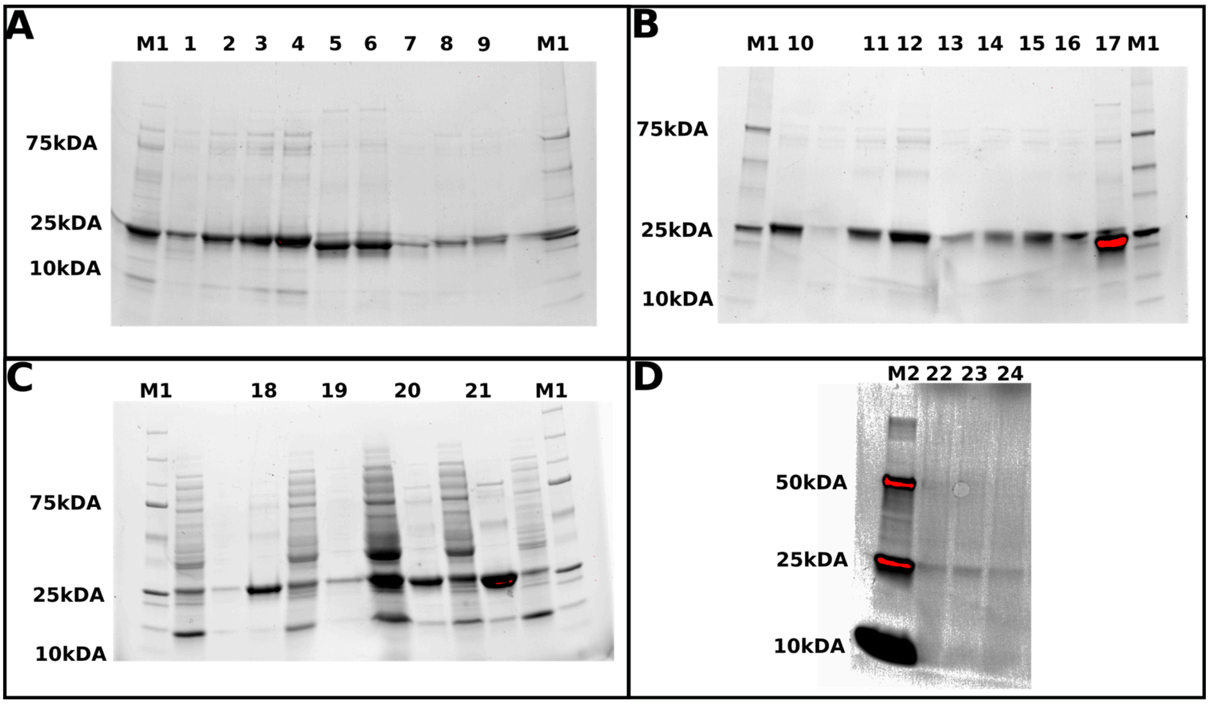

Supplementary Figure S7. **SDS PAGE gels for partially purified CAs (approx. 25.4 kDA). A. Wells showing M1 - Marker proteins, 1 - NtCA, 2 – N88K, 3 - N88H, 4 - K138L, 5 - Y144I, 6 - Y144L, 7 - A159D, 8 - A159F, 9 - D168P B. Wells showing M1 – Marker proteins, 10 – D168K, 11 – D197L, 12 – D197E, 13 – R210L, 14 – R210K, 15 – A159F_R210L, 16 – A159F_R210K, 17 – N88K_R210L. C. Wells showing M1 - Marker proteins, 18 – N88K_R210K 19 – D168K_R210L, 20 - D168K, R210K, 21 – Q1. D. M2 - Marker proteins, 22 – Q2, 23 – Q3 and 24 – Q4. M1 is the Precision Plus Protein™ Unstained Protein Standards, Strep-tagged from Bio-Rad and M2 is the Unstained Protein Standard, Broad Range (10-200 kDa) from New England Biolabs, Inc. Lanes in between numbered lanes in C have crude fractions before purification.**
